# Dietary Collagen Supplementation as a Strategy for Skin Health: A Narrative Review of Clinical Effects on Skin, Hair, Nails, and Wound Healing

**DOI:** 10.3390/nu18132141

**Published:** 2026-07-02

**Authors:** Angelika Biełach-Bazyluk, Marta Jurga, Iwona Flisiak, Edyta Zbroch

**Affiliations:** 1 Department of Dermatology and Venereology, Medical University of Bialystok, 15-540 Bialystok, Poland; marta.jurga9@gmail.com (M.J.); iwona.flisiak@umb.edu.pl (I.F.); 2 Department of Hypertensiology, Gastroenterology and Internal Medicine, Medical University of Bialystok, 15-540 Bialystok, Poland; edyta.zbroch@umb.edu.pl

**Keywords:** collagen peptides, skin aging, wound healing, hair disorders, nail disorders

## Abstract

Collagen is a key structural protein of the skin, essential for maintaining its mechanical strength, elasticity, and hydration. Oral collagen supplementation, particularly in the form of collagen peptides, has recently gained significant interest as a nutritional strategy to support skin health and overall wellbeing. However, the evidence regarding its effectiveness in supporting skin health and improving hair, nail, and wound-healing outcomes remains heterogeneous. The aim of this review is to summarize and critically evaluate the current human evidence on oral collagen supplementation and its potential role in supporting skin health, hair and nail quality, and wound healing. A targeted literature search was conducted using PubMed and Web of Science to identify clinical trials and relevant studies assessing the effects of collagen supplementation on skin aging parameters, including elasticity, wrinkles, hydration, and barrier function, as well as hair loss, nail disorders, and wound healing. Collagen-derived peptide supplementation has been associated with improvements in skin hydration, elasticity, wrinkle appearance, and dermal extracellular matrix organization, while also supporting hair thickness and strength, modestly enhancing nail growth, and promoting wound healing. Benefits are most consistent with low-molecular-weight hydroxyproline-rich peptides, with peptide characteristics appearing more important than collagen source. Evidence is limited by short study durations, heterogeneous designs, multi-ingredient formulations, and industry funding, which reduce confidence in the magnitude and consistency of the reported effects. Nevertheless, high-quality, long-term, independently funded trials with standardized outcomes are still required to confirm these findings.

## 1. Introduction

Beauty from within is a holistic, consumer-driven wellness and beauty trend characterized by the integration of nutrition-based interventions aimed at delaying skin aging and enhancing overall wellbeing. The trend has emerged from a growing preference for preventive strategies over corrective solutions in managing aging, alongside an increasing awareness of the role of diet and supplementation in influencing key biological processes such as oxidative stress and inflammation [1,2]. These processes are believed to play a significant role in the aging of both the skin and internal organs, suggesting that targeted nutritional interventions may help slow their progression. The rapid expansion of the nutraceutical market is reflected in the increasing use of nutricosmetics, defined as dietary supplements intended to improve skin appearance, particularly collagen-based products, whose popularity has risen significantly in recent years alongside a growing number of clinical studies investigating their effects on skin aging [3].

Collagen comprises a group of structural proteins and represents one of the most abundant protein families in the human body. To date, 28 types of collagen have been identified [4]. Among them, type I collagen is the most abundant and is primarily found in skin, bones, and tendons, while type II is predominant in cartilage and type III is commonly associated with extensible tissues such as skin and blood vessels [5]. The unique triple-helix structure of collagen provides high tensile strength and serves as the main structural component of the dermis, contributing to mechanical support, skin firmness, and elasticity [6]. Moreover, collagen plays a key role in wound healing and the formation of new extracellular matrix. It is not a passive component of the extracellular matrix but an active molecule that influences cell adhesion, migration, proliferation, and differentiation through interactions with specific cellular receptors [6]. In addition to the skin, collagen is a major structural component of bones, cartilage, ligaments, and tendons [5].

As age-related decrease in collagen content is considered a key hallmark of aging, many attempts have been made to slow its progression [7]. Due to its large molecular size, collagen is generally unable to penetrate the skin when applied topically, which limits the effectiveness of collagen-containing creams and lotions [8]. Gelatin, a common home-prepared product used for skin health, is a partially hydrolyzed form of collagen with a relatively high molecular weight and low solubility, which restricts its absorption and efficacy [9]. These limitations have driven increasing interest in nutraceutical approaches aimed at modulating skin biology from within, particularly collagen-based dietary supplements, which are proposed to influence dermal structure and function through systemic mechanisms.

Collagen-derived peptides, which are short chains of amino acids (typically 2–20) derived from collagen hydrolysis, have attracted considerable attention due to their high bioavailability and their potential to modulate biological processes [9]. In particular, experimental studies suggest that they may modulate cell signaling pathways, stimulate collagen synthesis, and regulate inflammation and extracellular matrix remodeling [10,11]. In dermatological cell culture models, collagen-derived peptides have been shown to stimulate dermal fibroblast proliferation and upregulate the expression of extracellular matrix components, including collagen, elastin, and hyaluronic acid, while modulating inflammatory signaling pathways [12,13,14].

In recent years, numerous studies have reported that supplementation with collagen-derived peptides in humans may exert anti-aging effects on the skin [15,16], be associated with improvements in the condition of hair and nails [17,18], and support wound healing processes [11,19]. However, several issues remain unresolved, including questions regarding their bioavailability [20], the source of collagen (animal versus marine) [21], and the reliability of existing studies, which are often limited by small sample sizes [22]. Furthermore, potential conflicts of interest related to industry funding, as well as regulatory challenges within the dietary supplement market, raise concerns about the reproducibility of these findings and their translation into consistent clinical outcomes [23,24].

**Figure 1:**
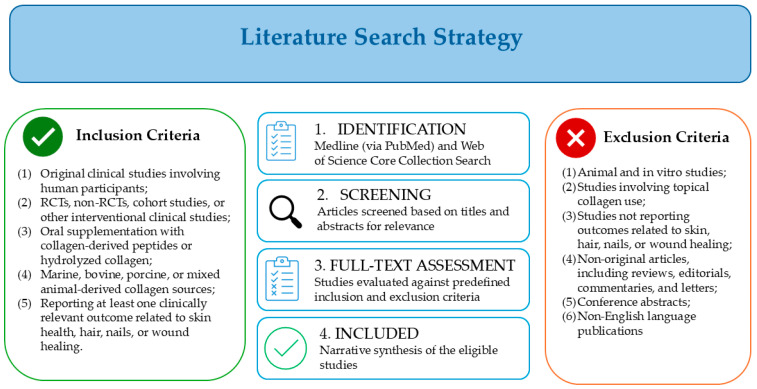
Overview of the literature search and study selection process. Abbreviations: RCTs—randomized controlled clinical trials; non-RCTs—non-randomized controlled clinical trials.

The purpose of this review is to summarize current evidence, highlight existing knowledge gaps, and propose future research directions on collagen supplementation as an approach to support skin and systemic wellbeing. We conducted a targeted literature search in MEDLINE (via PubMed) and the Web of Science Core Collection. In PubMed, Medical Subject Headings (MeSH) were applied, whereas in Web of Science, equivalent keyword searches were performed using the Topic (TS) field, which encompasses titles, abstracts, and keywords. The search strategy combined terms related to collagen supplementation, oral administration, and skin, hair, nails, and wound healing outcomes. Studies were included if they met the following criteria: (1) original clinical studies involving human participants; (2) randomized controlled trials, non-randomized controlled trials, cohort studies, or other interventional clinical studies; (3) oral supplementation with collagen-derived peptides or hydrolyzed collagen; (4) marine, bovine, porcine, or mixed animal-derived collagen sources; (5) reporting at least one clinically relevant outcome related to skin health, hair, nails, or wound-healing. Exclusion criteria comprised: (1) animal and in vitro studies; (2) studies involving topical collagen use; (3) studies not reporting skin, hair, nail, or wound-related outcomes; (4) non-original articles including reviews, editorials, commentaries, and letters; (5) conference abstracts; and (6) non-English language publications. Articles identified through the literature search were initially screened based on their titles and abstracts to assess their relevance to the objectives of this review. Studies considered potentially eligible were subsequently retrieved in full text and evaluated against the predefined inclusion and exclusion criteria. The final set of studies was synthesized narratively according to outcome domains, encompassing effects on skin structure, elasticity, hydration, wrinkle formation, photoaging-related outcomes, hair and nail quality, and wound healing, which are discussed in detail in the following sections. A schematic overview of the literature search strategy and study selection process is presented in Figure 1.

## 2. Pathophysiology of Skin Aging

The skin, as the external covering of the body, is the most accessible and observable indicator of age-related changes. According to current understanding, aging is characterized by a progressive decline in organ function, along with declining fertility and heightened vulnerability to disease, resulting from genetically determined, endogenous biological processes (intrinsic aging) and further influenced by environmental and lifestyle-related factors (extrinsic aging) [25].

Skin aging affects all layers of the skin and involves structural and functional alterations that correlate with clinical manifestations. In intrinsic aging, the epidermis typically becomes thinner, whereas photoaging may lead to epidermal thickening, particularly of the stratum corneum [26]. It is estimated that epidermal thickness decreases by approximately 6.4% per decade on average, accompanied by a corresponding reduction in the number of epidermal cells [27].

Age-related changes in the epidermis include reduced cellular turnover, impaired immune function, and alterations in pigmentation, contributing to a rougher skin surface and uneven skin appearance [28,29,30].

Decreased synthesis of the key structural components of the dermo-epidermal junction, including collagen types IV and VII, results in effacement of rete ridges and decreased surface area of contact between the epidermis and dermis [31]. Consequently, these changes increase skin fragility and may impair tissue homeostasis, contributing to greater vulnerability to age-related damage [32].

The dermis is the principal cutaneous compartment in which age-associated structural and molecular alterations occur [33]. Fibroblast senescence is a major driver of skin aging, resulting from both intrinsic aging associated with telomere shortening and extrinsic aging driven by increased oxidative stress and DNA damage [33,34]. Senescent fibroblasts exhibit reduced synthesis of collagen types I and III, elastin, and hyaluronic acid, leading to extracellular matrix disorganization, loss of dermal integrity, and the development of wrinkles, laxity, and reduced skin elasticity [28,32,33,35]. In addition, these cells acquire a senescence-associated secretory phenotype (SASP), characterized by increased production of pro-inflammatory mediators and matrix metalloproteinases, thereby promoting extracellular matrix degradation and chronic low-grade inflammation (inflammaging) [33,34,36,37].

Concurrently, age-related alterations in subcutaneous adipose tissue further contribute to loss of volume and structural support, exacerbating visible signs of skin aging [33,38].

Taken together, these multi-layered structural and functional alterations highlight the central role of extracellular matrix degradation, cellular senescence, and chronic inflammation in skin aging, thereby providing a strong rationale for interventions aimed at restoring dermal homeostasis, including collagen-derived peptides.

## 3. Collagen-Derived Peptides: Bioavailability and Mechanisms Relevant to Skin Biology

Dietary proteins and peptides are predominantly hydrolyzed into amino acids during digestion and subsequent metabolic processing in the circulation. However, accumulating evidence indicates that, following oral ingestion of collagen hydrolysate, a range of low-molecular-weight peptide species—particularly dipeptides and tripeptides—can evade complete proteolytic degradation and appear in the systemic circulation in intact forms [9,39]. Moreover, an animal in vivo model confirmed that low-molecular peptides may be efficiently transferred to the skin [39]. Dipeptides and tripeptides represent the most easily absorbed form of collagen-derived peptides, exhibiting higher intestinal uptake than larger peptide fragments present in collagen hydrolysates [9]. Among collagen-derived peptides, particular attention has been given to those containing 4-hydroxyproline (4Hyp) or 3-hydroxyproline (3Hyp), as the presence of these residues is thought to enhance resistance to proteolytic degradation, facilitate intestinal absorption, and thereby contribute to their increased biological activity [40,41].

Collagen-derived di- and tripeptides act as nutraceuticals, exhibiting anti-inflammatory, antioxidative, and immunomodulatory activities, in addition to modulating extracellular matrix turnover across multiple tissues [10,12,42]. Their anti-inflammatory effects are mediated through inhibition of the master regulator of inflammation, NF-κB, leading to decreased expression of pro-inflammatory mediators, including inducible nitric oxide synthase, cyclooxygenase-2 (COX-2), and the cytokines TNF-α, IL-1β, and IL-6 [10]. Collagen-derived peptides may enhance antioxidant defenses by upregulating key antioxidant enzymes and exert additional cytoprotective effects by attenuating lipid peroxidation and protecting cellular membranes [43].

**Figure 2:**
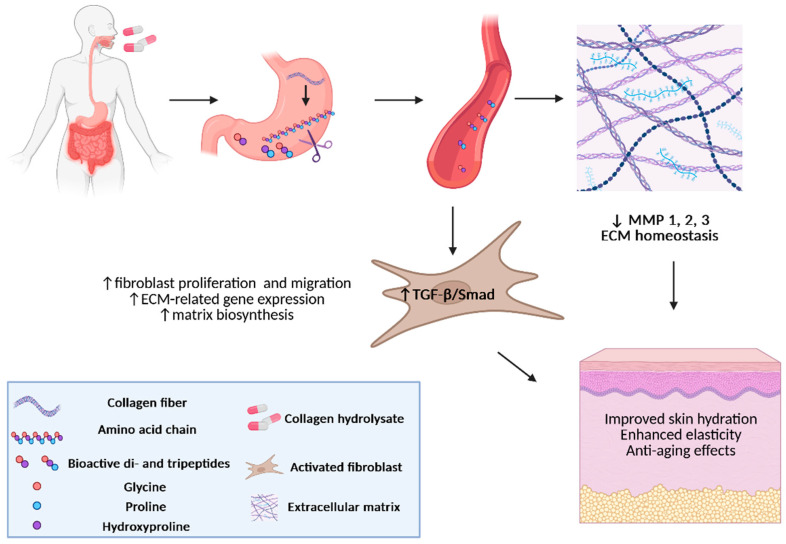
Overview of collagen peptide bioavailability and their role in fibroblast activation and extracellular matrix remodeling. Oral administration of collagen hydrolysates generates bioactive di- and tripeptides that resist complete proteolytic degradation and appear in intact form in systemic circulation. These peptides accumulate in dermal tissue, where they modulate extracellular matrix remodeling through activation of the TGF-β/Smad pathway, stimulation of fibroblast activity, enhancement of collagen and hyaluronic acid synthesis, and inhibition of matrix metalloproteinases, ultimately contributing to improved skin structure and function. Abbreviations: ECM—extracellular matrix, MMP—matrix metalloproteinase, TGF-β/Smad—transforming growth factor-β/Small Mothers Against Decapentaplegic signaling pathway. Own work based on [9,11,12,13,14,39,44,45,46,47,48]. Created in Biorender. Olivia Jakubowicz-Zalewska. (2026) (https://biorender.com/wp8pqzf, accessed on 29 June 2026).

A growing body of evidence highlights that collagen-derived peptides stimulate fibroblast proliferation and migration, activate the TGF-β/Smad pathway, and modulate MMP activity, thereby promoting extracellular matrix remodeling [11,12,13,14,19,44]. In particular, experimental studies suggest that they may increase the synthesis of collagen, elastin, hyaluronic acid, and versican through upregulation of related genes and suppression of MMP expression, including MMP-1, MMP-2, MMP-3, and MMP-12 [12,13,19,44,45,46,47,48]. Furthermore, they have been reported to modulate macrophage responses toward an anti-inflammatory M2-phenotype and may improve skin hydration [13,42,43,46]. Figure 2 summarizes the current understanding of the bioavailability of collagen-derived peptides and their proposed mechanisms of action relevant to skin biology.

Taken together, these mechanistic insights support the concept that orally administered collagen peptides may exert systemic biological activity extending beyond simple nutritional supplementation and may provide dermatological benefits.

## 4. Clinical Effects of Oral Collagen-Derived Peptides on Skin Aging

### 4.1. Overview of Clinical Evidence and Study Heterogeneity

In the following section, we discuss the current clinical evidence regarding collagen-derived peptide supplementation and skin health in humans. The available studies are highly heterogeneous with respect to peptide source, molecular weight distribution, and whether interventions consist of single-component collagen supplements or multicomponent nutraceutical formulations, which complicates direct cross-study comparisons.

Although clinical studies utilize collagen peptides derived from marine, bovine, porcine, and, less commonly, alternative sources, current evidence does not consistently demonstrate source-specific differences in clinical efficacy when matched for molecular weight and peptide composition. This observation is supported by a recent meta-analysis, which did not identify significant differences in outcomes according to collagen source [49]. Instead, bioactivity appears to be primarily influenced by peptide size distribution and hydroxyproline content. Clinical evidence suggests that specific peptide sequences, particularly prolylhydroxyproline (Pro-Hyp) and hydroxyprolylglycine (Hyp-Gly), are linked to more pronounced improvements in facial skin moisture, elasticity, wrinkle depth, and surface roughness [50]. In line with this, ultra-low-molecular-weight peptides (<1000 Da) have been associated with relatively rapid improvements in crow’s feet wrinkles in women [51].

Marine-derived collagen peptides are more extensively represented in clinical research compared with bovine or porcine sources. Consequently, they may appear to demonstrate more consistent clinical benefits; however, this likely reflects differences in study numbers and product availability rather than true source-dependent differences.

Nevertheless, pharmacokinetic data suggest that marine-derived collagen peptides, particularly those obtained from fish scales, may exhibit greater systemic exposure following oral ingestion compared with porcine skin-derived collagen hydrolysates, indicating potential differences in absorption and/or bioavailability between sources [20]. However, the clinical relevance of these pharmacokinetic differences remains unclear.

Beyond differences related to collagen source, novel delivery strategies have also been investigated to improve peptide bioavailability. One study evaluated oral disintegrating collagen films designed to enhance bioavailability through direct absorption across the oral mucosa while potentially bypassing gastrointestinal degradation [52]. It suggested that oral disintegrating collagen films may improve skin aging parameters, while ex vivo data indicate potentially higher mucosal absorption compared with conventional tablets. However, the clinical evidence remains preliminary due to the small, single-arm study design without a placebo-controlled comparison.

Overall, this heterogeneity should be considered when interpreting comparisons across studies.

### 4.2. Effects on Skin Hydration, Elasticity, and Wrinkle Formation

One notable example of a single-component intervention is the randomized, triple-blind, placebo-controlled study conducted by Evans et al. [53], which evaluated the effects of fish-derived hydrolyzed collagen peptides in women aged 45–60 years. After 12 weeks of supplementation, significant improvements in wrinkle reduction and skin elasticity were observed compared with placebo. Importantly, skin outcomes were assessed using objective instrumental methods, strengthening the reliability of the findings. However, improvements reported in subjective self-assessment measures were more pronounced than those observed with objective instrumental evaluations, and the potential risk of bias associated with industry funding should also be considered.

Similarly, the randomized clinical trial by Paula-Vieira et al. demonstrated that supplementation with single-ingredient bioactive collagen peptides for 12 weeks improved skin elasticity, hydration, and wrinkle parameters in middle-aged women, with stronger wrinkle reduction observed at the 10 g/day dose compared with 2.5 g/day [54]. A notable strength of the study compared with earlier collagen peptide trials was the inclusion of mechanistic biomarkers such as TGF-β and Klotho, suggesting potential immunomodulatory and regenerative effects beyond cosmetic outcomes.

The potential superiority of low-molecular-weight collagen peptides (<500 kDa) was demonstrated in several randomized, double-blind clinical trials [55,56,57,58,59]. These studies reported statistically significant improvements not only compared with placebo groups, but also when compared with conventional collagen hydrolysates used as comparators [55]. Participants receiving preparations enriched in hydroxyproline-containing low-molecular-weight collagen peptides achieved greater improvements in skin elasticity and dermal collagen content, as assessed using objective instrumental methods. Furthermore, enhanced intestinal absorption and bioavailability of preparations enriched with hydroxyproline-containing (X-Hyp) and hydroxyproline-glycine-containing (X-Hyp-Gly) peptide sequences were additionally supported by in vitro findings [55].

While most studies investigating marine-derived collagen utilize fish scales as the primary source, evidence also exists supporting the efficacy of fish cartilage-derived supplementation, which contains glycosaminoglycans and peptides, approximately 95% of which consist of low-molecular-weight fractions [60]. In a randomized, double-blind, placebo-controlled trial, it was demonstrated that improvements in overall skin structural quality were reported, suggesting that benefits may be achieved even at relatively lower supplementation doses.

Several studies additionally evaluated skin hydration using biophysical parameters, including transepidermal water loss (TEWL), as an indicator of epidermal barrier function. Notably, one randomized, double-blind, placebo-controlled trial incorporated adjustment for environmental confounders known to substantially influence skin hydration parameters, including ambient humidity, temperature, and ultraviolet exposure [61]. After adjustment for these climatic variables, supplementation with low-molecular-weight collagen tripeptides remained associated with a significant reduction in TEWL, suggesting beneficial effects on epidermal barrier function independent of seasonal environmental variation. Consistent with these findings, another study reported increased seasonal TEWL in the placebo group, whereas TEWL levels remained stable in the treatment group [62]. Improved TEWL following collagen peptide administration is suggested to result from raised levels of natural moisturizing factor in the stratum corneum [57,61,63].

Another noteworthy methodological approach was reported in a study that implemented a standardized skincare regimen prior to and throughout the intervention period, allowing the investigators to minimize variability related to cosmetic use [64]. Skin-related outcomes were assessed using standardized instrumental and dermatologist-evaluated methods, demonstrating significantly improved dermis density, skin hydration, elasticity, and wrinkle appearance over 84 days of supplementation. Similar to marine collagen peptides, bovine-derived products were shown to maintain skin improvements after a four-week washout period [65].

Data are also available for less commonly studied sources of collagen peptides. For instance, a randomized double-blind clinical trial enrolling 128 middle-aged women with signs of aging demonstrated statistically significant and instrumentally measured clinical improvement in skin elasticity, facial erythema and wrinkles after 12-week supplementation with hydrolyzed sternal chicken cartilage [66,67]. However, unlike several previous studies, this trial did not confirm significant reductions in TEWL [61,63,66].

### 4.3. Effects on Dermal Structure and Extracellular Matrix Remodeling

A randomized, triple-blind, placebo-controlled trial documented increased skin density and improved dermal structure, including higher collagen fiber content, following supplementation with a multi-ingredient collagen peptide dermonutrient over the study period, as assessed using confocal laser scanning microscopy [68]. These findings are further supported by a randomized placebo-controlled trial by Demir-Dora et al. [69], which reported improvements in skin elasticity, hydration, and dermal appearance after supplementation with hydrolyzed collagen peptides; however, the short follow-up period and female-only study population limit the broader generalizability of the results.

Mechanistic and ex vivo studies further suggest that some of the beneficial effects of bioactive collagen peptides may be mediated through stimulation of human dermal fibroblast proliferation, increasing the synthesis of procollagen type I, hyaluronic acid, and elastin, as well as modulating extracellular matrix turnover through decreased expression of MMP-1, MMP-3, MMP-12, and the release of TGF-β [14,46,48,70]. Furthermore, a pilot study indicated that collagen peptide intake increased plasma levels of insulin-like growth factor 1 (IGF-1) [62].

Additional evidence from a study investigating a multi-ingredient nutraceutical demonstrated increased serum fibronectin levels, reduced neutrophil elastase 2 activity associated with extracellular matrix degradation, and decreased levels of carbonylated proteins, a marker of oxidative damage [71]. Nonetheless, the interpretation of the findings is limited by several risk-of-bias concerns, including non-randomized or inadequately described study designs, potential industry involvement, use of multi-component formulations, and indirect biomarker interpretation [71].

### 4.4. Broader Dermatological Effects of Collagen Peptides

An emerging study by Lee et al. [72] suggested that improved skin texture is not solely attributable to wrinkle reduction; low-molecular-weight collagen peptides also significantly decreased sebum secretion and improved the appearance and measurable characteristics of facial pores. Most studies evaluate skin outcomes after 8–12 weeks of supplementation. However, clinical evidence from a small randomized trial suggests that even relatively short-term supplementation (4 weeks) with marine-derived collagen peptides may improve biomechanical skin properties associated with photoaging, particularly skin elasticity in sun-exposed areas [73].

Importantly, some studies suggest that certain favorable effects may persist after discontinuation of treatment, although the documented washout periods are relatively short [72,74]. In addition, one randomized controlled study incorporating paired pre- and post-intervention skin biopsies in a subgroup of participants reported histological improvement in solar elastosis following supplementation with a multi-ingredient collagen-based formulation [75].

Beyond hydration, low-molecular-weight collagen peptides have been hypothesized to influence broader skin biology, including elasticity biomechanics, inflammatory tone, and pigmentation-related pathways [76]. Although several reports have documented beneficial effects of collagen supplementation in photoaged populations, the clinical presentation of intrinsic aging and photoaging often overlaps, and the observed outcomes do not appear to differ substantially from those reported in other study cohorts [77].

Unlike many previous collagen studies focused exclusively on wrinkle reduction in older adults, Perin et al. additionally examined skin texture irregularities, redness, pigmentation homogeneity, acne-associated inflammation, and UV-induced erythema responses in younger women [78]. Supplementation with low-molecular-weight fish collagen peptides combined with L-cystine improved skin smoothness, reduced redness associated with acne lesions, and increased resistance to UV-induced erythema. However, the observed benefits cannot be attributed exclusively to collagen peptides, as L-cystine likely contributed substantially through its antioxidant, redox-regulating, and anti-inflammatory properties. In line with this, collagen peptide supplementation has also been associated with reductions in skin redness and pigmentation in a study including both male and female participants [79].

### 4.5. Effects of Collagen Peptides on Skin at Non-Facial Sites

Data on the impact of collagen supplementation on the skin beyond the face and neck are scarce and conflicting. For instance, low-molecular-weight marine collagen peptide supplementation significantly improved skin elasticity and reduced cellulite severity compared with placebo, with effects confirmed using objective instrumental assessment methods [80]. In contrast, collagen-derived peptides did not appear to improve photoaged skin at non-facial sites, as reported by Guadanhim et al. [81]. The study included postmenopausal women presenting with cutaneous atrophy, senile purpura, and stellate pseudoscars on the forearms, who were divided into subgroups receiving oral collagen, topical hydrolyzed collagen 2.5% serum or a combination of both interventions. The intervention lasted 6 months, and outcomes were evaluated using objective methods, including skin biopsies, cutometry, and ultrasound examination. Despite high rates of perceived improvement reported by participants, including enhanced skin hydration, smoothness, and wrinkle appearance, these subjective benefits were not supported by objective instrumental assessments, which failed to demonstrate significant measurable changes between the treatment groups [81].

Contrary, Nomoto and Iizaka reported significant improvements in stratum corneum hydration and skin elasticity following administration of an oral nutrition supplement containing 10 g of collagen peptides in hospitalized older adults [82]. Taken together, the available evidence regarding non-facial skin sites remains limited and somewhat inconsistent, precluding firm conclusions regarding the generalizability of these findings across different anatomical regions.

### 4.6. Multi-Ingredient Formulations and Combination Effects

The most common additional ingredients of nutraceuticals containing collagen peptides are antioxidants, including vitamins C and E, zinc, and selenium [83]. Overall, multi-ingredient formulations have been associated with outcomes generally consistent with those observed for pure collagen peptide supplementation in terms of skin elasticity, hydration, overall skin health, and anti-aging effects. However, the specific added value of antioxidants has not been clearly demonstrated [83]. Some authors have suggested that antioxidant-containing formulations may provide additional photoprotective effects and protection against environmentally induced skin damage [84]. Nevertheless, evidence supporting the antioxidant-related effects of such formulations remains inconclusive, as some studies failed to demonstrate improvements in key oxidative stress and glutathione-related biomarkers despite supplementation [85].

Subsequently, a randomized, double-blind, placebo-controlled trial evaluated both dermatological and musculoskeletal outcomes in adults presenting with visible signs of skin aging and osteoarthritis-related joint discomfort [75]. Participants received a multi-component oral formulation containing marine collagen peptides combined with antioxidants, vitamins, minerals, glucosamine, and chondroitin sulfate. Supplementation was associated with improvements in dermal histology, including enhanced collagen fiber organization and reduced solar elastosis, alongside reductions in joint discomfort, improved joint mobility, and enhanced self-perceived wellbeing. A notable strength of the study was the inclusion of histological skin biopsy assessment before and after intervention; however, these effects were observed with a combined formulation, suggesting potential synergistic effects rather than the isolated action of collagen alone.

The addition of hyaluronic acid to collagen peptide formulations has not consistently demonstrated additional clinical benefit over collagen peptides alone [86]. Nonetheless, another study described notable improvements in wrinkles, fine lines, skin elasticity, firmness, tone, texture, radiance, and overall skin quality following three months of oral supplementation with collagen peptides, hyaluronic acid, and micronutrients, with benefits confirmed by both expert assessment and participant self-reports [87]. However, these promising effects should be interpreted with caution due to the absence of a control group and lack of dietary monitoring.

Similarly, supplementation combining collagen peptides with coenzyme Q10 was associated with increased dermal density and improved wrinkle parameters and skin smoothness compared with placebo, while no significant effects were observed for skin hydration, transepidermal water loss, dermal thickness, or viscoelasticity [88].

Several botanical extracts, including djulis, were assessed for their efficacy and safety in combination with collagen peptides [89]. Overall, while the study reported comparable efficacy of the collagen–djulis formulation in improving key skin outcomes such as hydration, brightness, texture, and wrinkle appearance compared with similar interventions, its findings should be interpreted with consideration of limitations, including the modest sample size and reliance on indirect assessment methods for certain skin and collagen-related endpoints [89].

In contrast, collagen formulas enriched in methylsulphonylmethane, which is a naturally occurring organosulfur compound, were associated with favorable changes in certain structural skin parameters, including thickness and roughness [90]. However, methylsulphonylmethane alone does not consistently show strong anti-aging effects and most evidence of benefit comes from combination formulations or small trials [91].

One of the most notable studies evaluated the effects of marine-derived collagen peptides, administered alone or with calcium and vitamin D, on skin parameters, hair loss, and bone turnover in postmenopausal women [92]. The study was distinguished by a relatively long supplementation period of six months. Eighty participants were randomized into four groups: (1) calcium + vitamin D3 + collagen peptides, (2) collagen peptides alone, (3) calcium + vitamin D3, and (4) placebo. Improved skin hydration was observed in both collagen-supplemented groups, with greater improvement in the group additionally receiving calcium and vitamin D3 (23% vs. 5%). No significant changes in TEWL were detected, while skin elasticity improved similarly in both collagen groups. Interestingly, reduced hair shedding was reported across all active intervention arms. No significant effects on bone turnover biomarkers were observed, possibly due to low baseline bone turnover and the relatively short duration for detecting skeletal changes. The study is noteworthy for its double-blind design, absence of industry funding, and objective instrumental outcome assessment.

### 4.7. Safety Outcomes, Methodological Limitations and Risk of Bias

Across clinical studies, oral collagen-derived peptide supplementation appears to be well tolerated, with a very low incidence of adverse events reported. Safety outcomes have generally been comparable to placebo across different peptide sources, doses, and formulations, suggesting a favorable safety profile independent of product type.

Nevertheless, the evaluation of collagen safety extends beyond clinical tolerability and includes source-specific regulatory and quality considerations. Historically, concerns have been raised regarding the potential transmission of animal-derived pathogens, particularly bovine spongiform encephalopathy (BSE) associated with bovine-derived materials [93]. However, current manufacturing practices, regulatory oversight, and stringent requirements for raw material selection, traceability, and processing have substantially reduced these risks. In the European Union, collagen intended for human consumption must comply with specific hygiene and safety requirements regarding animal origin, processing conditions, and production controls [94].

Recent risk assessments indicate that collagen and gelatine produced according to current regulatory standards pose an extremely low risk of BSE transmission, supporting the effectiveness of existing control measures and manufacturing procedures [93]. Consequently, the safety of bovine- and porcine-derived collagen currently depends largely on compliance with stringent sourcing, traceability, and manufacturing requirements rather than on the animal origin itself. Nevertheless, marine-derived collagen has gained increasing attention due to consumer preferences, sustainability considerations, and its avoidance of concerns associated with terrestrial animal sources, including religious restrictions and historical concerns related to prion diseases [95].

From a safety perspective, marine collagen also requires appropriate quality control, particularly regarding potential allergenicity in individuals with fish or seafood allergies and the possible presence of environmental contaminants, including heavy metals [96]. Although collagen hydrolysis is generally considered to reduce allergenic potential through partial disruption of IgE-binding epitopes, the risk of allergic reactions cannot be considered negligible. Indeed, cases of severe allergic reactions, including anaphylaxis, following the ingestion of marine collagen hydrolysates have been reported in the literature [97]. Therefore, individuals with a history of fish allergy should use marine collagen-derived peptide supplements with caution, as residual IgE-reactive epitopes may persist despite extensive hydrolysis. However, studies indicate that effective purification processes and regulatory monitoring can minimize these risks, and commercially available collagen products must comply with applicable safety standards [96]. Overall, current evidence does not suggest major differences in safety profiles among approved collagen sources when appropriate quality control measures are applied, suggesting that product safety is determined primarily by raw material quality, traceability, purification procedures, and regulatory compliance rather than by collagen source alone.

Despite the generally favorable safety findings, the interpretation of both efficacy and safety outcomes is affected by several methodological limitations of the available clinical evidence. Several studies investigating collagen supplementation are limited by methodological weaknesses, including the absence of placebo controls, open-label designs, and small sample sizes [98]. Even though some studies enrolled a relatively large number of participants, conclusions are limited due to subgroup fragmentation, the absence of placebo control and blinding, concurrent cosmetic procedures, and reliance on subjective outcome measures [99]. An additional limitation observed in some studies is the use of branded commercial formulations, which raises concerns regarding potential sponsor-related bias and limits the generalizability of the results [99,100,101]. Furthermore, variability in outcome assessment methods and potential publication bias toward positive findings may further influence the interpretation of the results.

## 5. Clinical Effects of Oral Collagen-Derived Peptides on Hair and Nails

### 5.1. Biological Rationale for Collagen Peptides in Hair and Nail Health and Disease

Hair and nails are composed primarily of keratin rather than collagen, in contrast to the skin. While the relationship between collagen supplementation and hair or nail health may not be immediately obvious, several indirect mechanisms may help explain these effects. Collagen-derived peptides may affect the dermis and cellular processes involved in hair follicle and nail function [64,102]. Some peptides have also been shown to influence fibroblast activity and extracellular matrix remodeling [64]. In addition, amino acids derived from collagen may serve as substrates for keratin synthesis, potentially contributing to hair and nail structure. Experimental studies also indicate that collagen peptides may influence dermal papilla cell function and hair growth-related signaling pathways such as Wnt/β-catenin, although these effects are mainly demonstrated in preclinical models [103]. As a result, collagen supplementation has gained attention as a potential approach to support hair and nail health.

### 5.2. Clinical Evidence in Hair Disorders and Hair Quality

A prospective, comparative, randomized, blind study by Addor et al. also suggested a potential role of nutritional support in improving hair condition, particularly in women with telogen effluvium [98]. Two oral supplements were compared over 90 and 180 days. Both groups showed improvements in parameters related to hair loss, volume, density, shine, strength, and overall hair quality. However, the formulation containing a broader combination of nutrients showed earlier effects, with significant improvements already visible after 90 days. By 180 days, both groups had improved, although the same formulation continued to show slightly better results. Digital trichoscopy also revealed a significant increase in mean hair density in the group receiving the broader nutrient formulation. These observations support the idea that nutritional factors may contribute to idiopathic telogen effluvium and that supplementation may potentially support hair recovery. A key limitation of the study is the absence of a placebo-controlled group, as both groups received active supplementation, which limits the ability to attribute observed effects to the intervention itself.

Gwon et al. conducted a randomized, double-blind, placebo-controlled trial to assess the effects of low-molecular-weight collagen peptides on hair health in adults with damaged hair [17]. Compared to placebo, the supplemented group showed improvements in several objective and subjective parameters, including hair shine, strength, thickness, and density. Improvements were seen not only in measured hair parameters but also in hair shaft structure, confirmed by scanning electron microscopy (SEM) evaluation of surface integrity. The findings point to a beneficial effect of low-molecular-weight collagen peptides on overall hair quality and strength, although the study involved individuals with damaged hair rather than patients with clinical hair loss [17].

In line with this, in a randomized, placebo-controlled study involving 114 women over 24 weeks, low-molecular-weight collagen peptides (LMWCPs) were evaluated for their broader effects on skin and hair parameters. Women taking 1000 mg daily showed improvements in hair thickness compared to the placebo group. A larger proportion of participants in the collagen group also showed measurable hair thickening during the study period. Overall, these findings suggest that low-molecular-weight collagen peptides may be associated with changes in hair health parameters, although the contribution of enhanced absorption and bioavailability requires further confirmation [80].

Milani et al. explored whether collagen-based supplementation could benefit individuals with hair loss conditions such as androgenetic alopecia or telogen effluvium [102]. In this 12-week randomized controlled trial involving 83 participants with androgenetic alopecia/female androgenetic alopecia (AGA/FAGA) or chronic telogen effluvium (TE), those who received a supplement containing hydrolyzed collagen, amino acids, iron, and selenium alongside standard treatment showed greater improvements in hair growth parameters compared to those receiving treatment alone. The findings suggest that collagen-based supplementation may provide additional benefits when combined with standard therapies; however, the specific contribution of collagen peptides remains uncertain and requires further investigation.

The main clinical studies evaluating oral collagen supplementation for hair and nail health are summarized in Table 1.

### 5.3. Clinical Evidence for Nail Health

Evidence regarding the effects of collagen supplementation on nail health remains limited and is considerably less well established compared with the more extensively studied outcomes related to skin and hair. So far, one study has examined the effects of collagen supplementation on brittle nails, reporting improvements in nail growth and reduced brittleness [18]. In a 24-week study involving 25 women, daily supplementation with 2.5 g of bioactive collagen peptides was associated with an approximately 12% increase in nail growth and a 42% reduction in the frequency of broken nails. In addition, 64% of participants reported an overall improvement in nail condition, and this percentage increased to 88% four weeks after supplementation ended, suggesting that some benefits may persist beyond the supplementation period, although this finding requires confirmation in larger controlled studies. Overall, the findings suggest that collagen peptides may have a potential role in supporting nail growth and strength, although larger controlled studies are still needed [18].

Similar nail-related effects have also been reported in studies primarily investigating skin aging. In a double-blind, placebo-controlled trial involving 85 East Asian women aged 43–65, daily supplementation with 5 g of collagen peptides for 12 weeks led to visible improvements in nail appearance, including reduced yellowness and improved brightness [64].

### 5.4. Summary, Limitations and Strength of Evidence

Overall, the available clinical evidence suggests potential beneficial effects of collagen peptide supplementation on certain aspects of hair quality, including thickness, strength, and structural integrity; however, the number of well-controlled clinical studies remains limited, and findings are based on relatively small and heterogeneous study populations. Evidence regarding nail health is even more scarce, with only a few controlled trials reporting modest improvements in nail growth and brittleness, and the overall certainty of the evidence is constrained by short study durations, variability in study design, and the frequent absence of long-term follow-up data.

## 6. Clinical Effects of Oral Collagen-Derived Peptides on Wound Healing

### 6.1. Biological Rationale for Collagen in Wound Healing

Wound healing is a highly coordinated, multistep biological process consisting of four overlapping phases: hemostasis, inflammation, proliferation, and tissue remodeling. Proper wound healing depends on interactions between many factors, such as inflammatory cells, fibroblasts, growth factors, and extracellular matrix components. Initially, vascular constriction and platelet aggregation help limit bleeding and form a temporary barrier at the site of injury. The inflammatory phase is associated with the removal of cellular debris and bacteria, as well as the release of cytokines and growth factors that regulate further repair mechanisms. Increased vascular permeability and migration of inflammatory cells into the wound area also occur at this stage [104,105].

Beyond the classical inflammatory response, recent studies highlight the role of specialized pro-resolving mediators (SPMs), bioactive lipid mediators derived from polyunsaturated fatty acids (PUFAs), including lipoxins, resolvins, protectins, neuroprotectins, and maresins. Rather than simply suppressing inflammation, these mediators actively promote the resolution phase of inflammation, facilitating the transition from inflammatory processes to tissue repair and regeneration [106,107].

During the proliferative phase, fibroblasts synthesize collagen and other extracellular matrix components that contribute to granulation tissue formation and tissue repair. Re-epithelialization and neovascularization also take place during this stage. Finally, the remodeling phase involves collagen reorganization and scar maturation, leading to increased tissue strength over time [104,105].

Collagen plays a central role throughout the wound healing process by providing structural support for newly formed tissue and facilitating cell migration, angiogenesis, and wound contraction [108,109]. As healing continues, collagen fibers gradually reorganize and contribute to the maturation of the repaired tissue [108].

Experimental studies further suggest that collagen-derived peptides may stimulate fibroblast proliferation, collagen synthesis, elastin production, and cell migration, thereby supporting tissue repair processes. For example, a human collagen type I alpha-2-derived peptide increased collagen type I synthesis and accelerated wound closure in cultured human dermal fibroblasts in vitro [109].

Taken together, these findings have contributed to growing interest in oral collagen supplementation as a potential supportive strategy for wound healing and tissue repair.

### 6.2. Clinical Evidence in Chronic and Acute Wounds

Emerging clinical evidence further suggests that collagen-derived peptide supplementation may be associated with improved wound healing outcomes in selected patient populations. One of the key studies supporting the role of oral collagen supplementation in wound healing was a randomized controlled trial conducted by Lee et al. The trial evaluated the effects of a fortified collagen protein hydrolysate administered three times daily on Pressure Ulcer Scale for Healing (PUSH) scores, a validated tool used to assess pressure ulcer healing over time, in patients with stage II–IV pressure ulcers. Participants were randomized to receive either standard care plus collagen supplementation or standard care plus placebo. After 8 weeks, the supplementation group showed greater improvement in PUSH scores compared with the placebo group, suggesting a potential beneficial effect of collagen supplementation on pressure ulcer healing. Nevertheless, the study had several limitations, including the relatively small sample size, short intervention period, and the inclusion of only long-term care residents [110].

Additional evidence was provided by a later double-blind, multi-centric, placebo-controlled randomized trial conducted in patients with stage II–III pressure ulcers. In this study, two collagen hydrolysate formulations were compared with placebo over a 16-week period. The formulation containing higher levels of the bioactive dipeptides Pro-Hyp and Hyp-Gly significantly improved PUSH scores and Pressure Sore Status Tool (PSST) scores, another validated measure of pressure ulcer healing, as well as wound area compared with placebo, whereas the formulation with lower dipeptide content showed improvement mainly in PUSH scores. These results suggest that differences in peptide composition may affect wound-healing outcomes [111].

Another area of research has explored the potential role of collagen-derived peptides in burn wound healing. For example, in a randomized clinical trial involving patients with burns covering 20–45% of total body surface area, supplementation with 40 g/day of collagen hydrolysate, either alone or combined with omega-3 fatty acids, was associated with faster wound healing compared with placebo [112]. Both collagen-treated groups showed shorter times to achieve 95% wound healing and complete wound closure, as well as improved Vancouver Scar Scale scores. However, no significant differences were observed between the collagen-only and collagen plus omega-3 groups, suggesting no clear additional benefit of omega-3 co-supplementation within the study period [112].

Similar findings were reported in a randomized double-blind pilot clinical trial conducted in men with burns covering 20–30% of total body surface area, which evaluated the effects of a hydrolyzed collagen-based supplement administered for 4 weeks. Patients receiving collagen supplementation showed faster wound healing and higher serum pre-albumin concentrations compared with the control group. Hospital stay also tended to be shorter in the collagen group, although the difference did not reach statistical significance. The study was limited by the relatively small sample size and the inclusion of only male participants [113].

More limited evidence comes from a study by Choi et al. [108], which investigated the effects of oral collagen tripeptide on wound healing and skin recovery using both in vitro models and a small clinical pilot study. In vitro experiments demonstrated enhanced cell migration and accelerated wound closure following collagen treatment, although no clear dose–response relationship was identified. In contrast, collagen supplementation did not significantly increase collagen gel contraction compared with controls. The clinical pilot study included eight healthy women undergoing fractional photothermolysis, of whom four received oral collagen supplementation. Post-laser erythema appears to resolve more rapidly in the supplementation group, which also showed greater recovery of skin hydration and improved post-treatment skin elasticity compared with controls. Nevertheless, interpretation of these findings is limited by the small sample size, short study duration, and the fact that the intervention was evaluated in a dermatological procedure model rather than in patients with clinical wounds [108]. The key clinical studies evaluating oral collagen supplementation and related nutritional interventions in wound healing are summarized in Table 2.

### 6.3. Indirect Nutritional Support of Wound Healing

In addition to direct collagen supplementation, several studies have investigated nutritional interventions that may indirectly support wound healing by modulating processes involved in collagen synthesis and extracellular matrix formation. In elderly individuals, oral arginine supplementation was associated with enhanced wound healing by increasing collagen deposition and total protein content in healing tissue [114]. Arginine supplementation was also associated with improved lymphocyte activity and increased IGF-1 levels [114].

Similarly, a randomized controlled trial investigated perioperative multinutrient supplementation in men undergoing inguinal hernia repair. The intervention, containing arginine, glutamine, vitamin C, and zinc, increased wound-fluid concentrations of type I procollagen propeptide during early wound healing, suggesting enhanced collagen synthesis. However, no significant reduction in epidermal healing time was observed. Importantly, the study did not directly evaluate oral collagen supplementation, and therefore its findings cannot be directly extrapolated to collagen peptide supplementation [115].

### 6.4. Summary, Limitations and Strength of Evidence

To sum up, clinical evidence suggests that oral collagen-derived peptide supplementation may support wound healing outcomes in selected patient populations, particularly in pressure ulcers and burn injuries. Reported benefits include improvements in validated wound-healing scores and accelerated wound closure in some studies. However, the current evidence base remains limited by small sample sizes, heterogeneity in peptide formulations and dosing regimens, variability in study populations, and frequent inclusion of adjunct nutritional components. Consequently, while mechanistic and early clinical findings are promising, definitive conclusions regarding the magnitude and specificity of the effects of oral collagen supplementation on wound healing cannot yet be established.

## 7. Conclusions

The growing interest in collagen-derived peptides reflects a broader shift toward preventive and nutrition-based approaches to healthy aging. Experimental evidence suggests that orally administered collagen peptides may influence multiple biological pathways involved in skin aging, including extracellular matrix remodeling, fibroblast activity, oxidative stress, and low-grade inflammation, thereby providing a mechanistic basis for their proposed dermatological benefits.

Overall, current clinical evidence suggests that oral collagen-derived peptide supplementation may be associated with favorable changes in several parameters related to skin aging, particularly skin hydration, elasticity, wrinkle appearance, and dermal extracellular matrix organization. Furthermore, it may have potential benefits for hair parameters, including increased thickness, strength, and structural integrity, as well as for nails, for which modest improvements in growth and reduced brittleness have been reported in limited studies. In addition, preliminary evidence suggests possible positive effects on wound healing, with studies reporting improved healing scores and shorter time to wound closure in selected conditions, such as pressure ulcers and burn injuries.

The most consistent benefits have been reported for low-molecular-weight hydroxyproline-rich peptides across different collagen sources. Collagen source appears to be a secondary determinant of efficacy compared with peptide molecular characteristics.

Despite the growing body of evidence supporting the beneficial effects of collagen-derived peptides on skin aging, hair and nail health, and wound healing, several important knowledge gaps remain. Specifically, substantial heterogeneity in peptide composition and molecular weight, dosing regimens, study populations, outcome assessment methods, and the frequent use of multi-component formulations limit definitive conclusions regarding the magnitude and specificity of these effects. Most available clinical trials evaluate relatively short intervention periods, typically ranging from 8 to 12 weeks. Consequently, the long-term efficacy and durability of benefits after supplementation discontinuation remain insufficiently investigated.

Moreover, outcome measures vary considerably across studies, ranging from subjective assessments to instrumental evaluations, reducing comparability between trials and complicating quantitative interpretation of the available evidence. This is particularly evident in hair and nail studies, where outcomes are often based on heterogeneous subjective scoring systems and non-standardized assessments. In contrast, wound healing studies more frequently employ validated scales; however, the evidence base remains limited, with small sample sizes, predominantly pilot or early-phase designs, and considerable variability in wound types, including pressure ulcers, burns, and other chronic or acute lesions. Several studies assessing hair, nails, and wounds are limited by methodological weaknesses, including lack of placebo control or incomplete blinding, insufficient reporting of randomization and allocation procedures.

Another important limitation is the predominance of studies on skin aging involving healthy middle-aged women. In contrast, studies on hair, nails, and wound healing exhibit considerable heterogeneity in both clinical indications and outcome assessment tools, limiting cross-study comparability.

In addition, many studies evaluate multi-ingredient formulations, making it difficult to determine the specific effects attributable to collagen peptides alone. Furthermore, a substantial proportion of these studies are industry-funded and assess proprietary commercial products, which may raise concerns regarding the reliability and generalizability of the findings, as well as the potential for bias.

Consequently, there is a need for large-scale, independently funded randomized controlled trials directly comparing pure collagen peptides with formulations containing additional vitamins and antioxidant compounds, incorporating longer follow-up periods and standardized, objective outcome assessment methods. In the context of skin aging studies, greater age and sex diversity among participants would also improve the applicability of the results to broader populations.

Taken together, current evidence suggests that collagen-derived peptides may represent a promising supportive strategy for skin health and wound healing, although high-quality, long-term clinical confirmation is still required.

## Figures and Tables

**Table 1:** Summary of clinical studies evaluating collagen supplementation for hair and nail health.

Author	Year	Population/Model	Intervention	Comparator	Main Findings	Limitations
Evidence on hair outcomes						
Addor et al. [98]	2018	Women with telogen effluvium	Two oral nutritional supplements	Active comparator (no placebo)	Hair loss decreased, with improvements in density and overall hair quality observed over the 6-month period.	Multiple ingredients; no placebo
Gwon et al. [17]	2026	Adults with damaged hair	Low-molecular-weight collagen peptides	Placebo	Hair density, strength, shine, and structure improved over time	Findings limited to individuals with damaged hair
Hwang et al. [80]	2026	Women with cellulite	1000 mg low-molecular-weight collagen peptides daily	Placebo	Hair thickness increased after 24 weeks of supplementation	Hair thickness assessed as a secondary outcome
Milani et al. [102]	2023	Patients with hair loss (AGA, FAGA, or TE)	Hydrolyzed fish collagen + amino acids, iron, selenium + standard treatment	Standard treatment alone	Better outcomes when the supplement was added to standard treatment	Adjunctive supplementation; effect of collagen alone cannot be isolated
Evidence on nail outcomes						
Hexsel et al. [18]	2017	Women with brittle nails	2.5 g bioactive collagen peptides daily	No control group	Nails grew faster and were less prone to breaking	Small uncontrolled study
Vleminckx et al. [64]	2024	Healthy women	5 g collagen peptides daily	Placebo	Reduced nail yellowness after supplementation	Nail parameters were evaluated together with skin outcomes

Abbreviations AGA—androgenetic alopecia; FAGA—female androgenetic alopecia; TE—telogen effluvium.

**Table 2:** Key clinical studies evaluating oral collagen supplementation and related nutritional interventions in wound healing.

Study	Year	Wound Type	Population	Design/Intervention	Comparator	Duration	Main Outcomes	Main Findings
Alipoor et al. [112]	2023	Major burns	*n* = 66 burn patients (20–45% TBSA)	RCT; Beverage containing 40 g/day collagen or 40 g/day collagen + 3 g/day omega-3 fatty acid	Placebo beverage	4 weeks	Wound healing rate, Vancouver scar scale (VSS), hospital stay, metabolic biomarkers (FGF-21, adiponectin, TGF-β1)	Both collagen groups showed faster healing and better VSS than placebo; no clear additional wound benefit from omega-3
Bagheri et al. [113]	2020	Burn wounds	*n* = 31 men with burns (20–30% TBSA)	RCT; Collagen-based supplement (1000 kcal) + standard care	Isocaloric placebo + standard care	4 weeks	Serum pre-albumin, rate of wound healing, length of hospital stay, and anthropometries	Collagen supplementation significantly increased pre-albumin levels, accelerated wound healing (HR 3.7), and clinically reduced hospital stay compared with placebo
Choi et al. [108]	2014	Skin recovery after fractional photothermolysis	*n* = 8 healthy women	Pilot RCT; 3 g/day oral collagen peptide	Control group	4 weeks	Erythema index, TEWL, skin hydration, skin elasticity	Faster post-laser erythema resolution, faster recovery of skin hydration, and improved post-treatment skin elasticity compared with controls.
Kirk et al. [114]	1993	Experimental model: subcutaneous catheter-induced wound and split-thickness skin wound	*n* = 30 healthy adults aged >65 years	Prospective randomized trial; Supplements of 30 g of arginine aspartate daily (17 g of free arginine)	Placebo syrup	14 days	Collagen deposition, total protein content, rate of wound epithelialization	Increased collagen deposition and protein content; no difference in epithelialization time
Kjaer et al. [115]	2020	Early surgical wound repair	*n* = 21 men undergoing inguinal hernia repair	RCT; 14 g L-arginine, 14 g L-glutamine, 1250 mg vitamin C, and 55 mg zinc daily + high-quality protein (1.5 g protein/kg)	High-quality protein (1.5 g protein/kg)	28 days	Biomarkers of collagen synthesis: CICP, PRO-C3, and PRO-C5 in the sera and in the wound fluids, epidermal healing time	Increased early collagen synthesis biomarkers; no significant effect on epidermal healing time
Lee et al. [110]	2006	Pressure ulcers	*n* = 89 residents with stage II–IV pressure ulcers	RCT; Concentrated fortified collagen protein hydrolysate supplement + standard care, 3 times daily	Placebo + standard care 3 times daily	8 weeks	Pressure Ulcer Scale for Healing (PUSH)	Improved PUSH scores and approximately twice the rate of pressure ulcer healing compared with placebo
Sugihara et al. [111]	2018	Pressure ulcers	*n* = 120 adults with stage II–III pressure ulcers	RCT; Standard care plus one of two types of collagen hydrolysate CH-a, which contained low levels of Pro-Hyp and Hyp-Gly, and CH-b, containing high levels of Pro-Hyp and Hyp-Gly	Placebo	16 weeks	PUSH, Pressure Sore Status Tool (PSST), wound area	CH-b improved PUSH, PSST, and wound area versus placebo; CH-a improved PUSH only

Abbreviations CH-a, collagen hydrolysate a; CH-b, collagen hydrolysate b; CICP, type I collagen propeptide; FGF-21, fibroblast growth factor-21; HR, hazard ratio; Hyp-Gly, hydroxyprolylglycine; PRO-C3, type III procollagen propeptide; PRO-C5, type V procollagen propeptide; Pro-Hyp, prolylhydroxyproline; PSST, Pressure Sore Status Tool; PUSH, Pressure Ulcer Scale for Healing; RCT, randomized controlled trial; TBSA, total body surface area; TEWL, transepidermal water loss; TGF-β1, transforming growth factor beta-1; VSS, Vancouver Scar Scale.

## Data Availability

No new data were created or analyzed in this study. Data sharing is not applicable to this article.

## Abbreviations

3Hyp	3-hydroxyproline
4Hyp	4-hydroxyproline
AGA	androgenetic alopecia
BSE	bovine spongiform encephalopathy
CH-a	collagen hydrolysate with low levels of prolylhydroxyproline (Pro-Hyp) and hydroxyprolylglycine (Hyp-Gly)
CH-b	collagen hydrolysate with high levels of Pro-Hyp and Hyp-Gly
CICP	type I collagen propeptide
COX-2	cyclooxygenase-2
ECM	extracellular matrix
FAGA	female androgenetic alopecia
FGF-21	fibroblast growth factor-21
GLY	glycine-containing dipeptide (Hydroxyprolylglycine, Hyp-Gly)
HR	hazard ratio
Hyp-Gly	hydroxyprolylglycine
IGF-1	insulin-like growth factor-1
IL	interleukin
IL-1β	interleukin-1 beta
IL-6	interleukin-6
IL-8	interleukin-8
LMWCP	low-molecular-weight collagen peptide
MeSH	Medical Subject Headings
MMP	matrix metalloproteinase
NF-κB	nuclear factor kappa B
PRO-C3	type III procollagen propeptide
PRO-C5	type V procollagen propeptide
Pro-Hyp	prolylhydroxyproline
PSST	Pressure Sore Status Tool
PTFE	polytetrafluoroethylene
PUFAs	polyunsaturated fatty acids
PUSH	Pressure Ulcer Scale for Healing
RCT	randomized controlled trial
SASP	senescence-associated secretory phenotype
SEM	scanning electron microscopy
SPMs	specialized pro-resolving mediators
TBSA	total body surface area
TE	telogen effluvium
TEWL	transepidermal water loss
TGF-β	transforming growth factor beta
TGF-β/Smad	transforming growth factor-β/Small Mothers Against Decapentaplegic signaling pathway
TNF-α	tumor necrosis factor alpha
TS	Topic field
VSS	Vancouver Scar Scale
Wnt/β-catenin	Wingless/Integrated beta-catenin signaling pathway
